# IKKε inhibits PKC to promote Fascin-dependent actin bundling

**DOI:** 10.1242/dev.138495

**Published:** 2016-10-15

**Authors:** Tetsuhisa Otani, Yosuke Ogura, Kazuyo Misaki, Takuya Maeda, Akiyo Kimpara, Shigenobu Yonemura, Shigeo Hayashi

**Affiliations:** 1 Laboratory for Morphogenetic Signaling, RIKEN Center for Developmental Biology, Kobe, Hyogo 650-0047, Japan; 2 Electron Microscope Laboratory, RIKEN Center for Developmental Biology, Kobe, Hyogo 650-0047, Japan; 3 Department of Biology, Kobe University Graduate School of Science, Kobe, Hyogo 657-8501, Japan

**Keywords:** Actin, Fascin, Singed, IKKε, PKC, Bristle morphogenesis, *Drosophila*

## Abstract

Signaling molecules have pleiotropic functions and are activated by various extracellular stimuli. Protein kinase C (PKC) is activated by diverse receptors, and its dysregulation is associated with diseases including cancer. However, how the undesired activation of PKC is prevented during development remains poorly understood. We have previously shown that a protein kinase, IKKε, is active at the growing bristle tip and regulates actin bundle organization during *Drosophila* bristle morphogenesis. Here, we demonstrate that IKKε regulates the actin bundle localization of a dynamic actin cross-linker, Fascin. IKKε inhibits PKC, thereby protecting Fascin from inhibitory phosphorylation. Excess PKC activation is responsible for the actin bundle defects in *IKKε*-deficient bristles, whereas PKC is dispensable for bristle morphogenesis in wild-type bristles, indicating that PKC is repressed by IKKε in wild-type bristle cells. These results suggest that IKKε prevents excess activation of PKC during bristle morphogenesis.

## Abstract (precis)

**Summary:** The protein kinase IKKϵ is active at the growing tip of *Drosophila* bristles and prevents excess PKC activation during bristle actin bundle organization and morphogenesis.

## INTRODUCTION

*Drosophila* bristles are external sensory organs involved in mechanosensation and chemosensation that are formed by the elongation of single cells during the pupal stage (Lees and Waddington, 1942; Tilney and DeRosier, 2005). We and others have been investigating the role of a serine threonine kinase, IκB kinase ε (IKKε, also known as Ik2), in bristle cell morphogenesis (Oshima et al., 2006; Shapiro and Anderson, 2006; Koto et al., 2009; Bitan et al., 2010; Otani et al., 2011, 2015). IKKε is locally activated at the tip of growing bristles, and acts as a signaling center for bristle cell elongation by coordinately regulating cytoskeleton organization and vesicle trafficking (Bitan et al., 2010; Otani et al., 2011, 2015). IKKε regulates the shuttling movement of recycling endosomes through phosphorylation of the Rab11 effector molecule Nuclear fallout (Nuf) (Otani et al., 2011). Inhibition of Nuf can restore the Rab11 accumulation phenotype in *IKKε*-deficient bristles, but does not rescue the actin bundle phenotype, suggesting that IKKε regulates actin bundle organization through a distinct signaling pathway that remains to be characterized (Otani et al., 2011).

In *Drosophila* bristles, parallel actin bundles run beneath the cellular cortex throughout the bristle shaft (Overton, 1967; Appel et al., 1993) and are assembled through the sequential action of two actin-bundling proteins: Forked and Fascin (also known as Singed) (Tilney et al., 1995, 1996, 1998; Wulfkuhle et al., 1998). Forked initially bundles the newly generated actin filaments in the tip region, whereas Fascin subsequently promotes the hexagonal paracrystalline packing of actin filaments (Tilney et al., 1995, 1996, 1998). The loss of either Forked or Fascin results in disorganized actin bundles accompanied by a gnarled morphology of the bristles, suggesting that the proper cross-linking of actin filaments is essential to maintain the morphology of the bristles (Lees and Waddington, 1942; Lees and Picken, 1944; Overton, 1967; Cant et al., 1994; Petersen et al., 1994; Tilney et al., 1995).

In this study, we sought to understand how IKKε regulates actin bundle organization in bristle morphogenesis. We demonstrate that IKKε regulates actin bundle organization by promoting Fascin-dependent actin bundling. Mechanistically, IKKε inhibits PKC, thereby protecting Fascin from PKC-dependent inhibitory phosphorylation. Interestingly, although excess PKC activation is responsible for actin bundling defects in *IKKε*-deficient bristles, PKC loss of function in a wild-type background yields no visible phenotype, suggesting that PKC activity is repressed in wild-type bristles by the action of IKKε. These results suggest that IKKε prevents undesired PKC activation during bristle morphogenesis.

## RESULTS

### IKKε regulates Fascin localization

**Fig. 1.**
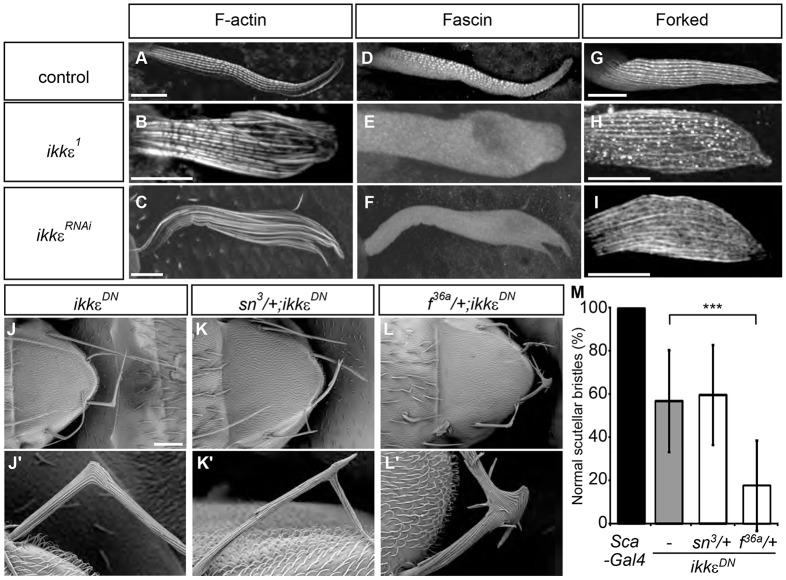
**IKKε regulates Fascin.** (A-C) Phalloidin staining of bristles. The parallel organization of actin bundles is disorganized in *IKKε*-deficient bristles. (D-F) Anti-Fascin antibody staining of bristles. Fascin localization along the actin bundles was severely reduced in *IKKε*-deficient bristles. (G-I) Anti-Forked antibody staining of bristles. Forked localization along the actin bundles was not perturbed in *IKKε*-deficient bristles. (J-M) Genetic interactions between *IKKε*, *fascin* [*singed* (*sn*)] and *forked* (*f*). (J-L′) SEM analysis shows that the reduction of *forked* significantly exaggerates the bristle morphology phenotype of *IKKε^DN^* bristles. Representative bristles are shown at higher magnification in J′-L′. (M) Quantification of the bristle morphology phenotype. The percentage of normal scutellar bristles is shown as mean±s.d. *n*=30 each. ***P<1.0×10^−8^ by two-tailed *t-*test. Scale bars: 10 µm in A-I; 100 µm in J-L. See also Figs S1 and S4.

Phalloidin staining of thorax macrochaetes revealed that actin bundles run in parallel along the long axis of developing bristles (Fig. 1A). The actin bundle organization was severely disorganized when IKKε is inactivated through mutation (*IKKε^1^* or *IKKε^alice^*, strong loss-of-function alleles with missense mutations in the kinase domain), RNAi (*IKKε^RNAi^*), or the expression of dominant-negative IKKε (*IKKε^DN^*; IKKε[K41A], a point mutation in the ATP-binding loop) (Fig. 1B,C, Fig. S1A,B) (Bitan et al., 2010; Otani et al., 2011). Antibody staining revealed that Forked and Fascin, the two actin-bundling proteins involved in bristle morphogenesis, are localized along actin bundles in control bristles (Fig. 1D,G) (Cant et al., 1994; Petersen et al., 1994), but show discontinuous staining, which is most likely due to poor penetration of antibodies into the tightly packed actin bundles (∼8 nm distance between actin filaments when bundled by Fascin; Jansen et al., 2011). Intriguingly, Fascin localization on actin bundles was severely decreased in *IKKε*-deficient bristles (Fig. 1E,F, Fig. S1C,D). Nevertheless, cytoplasmic Fascin signals were detected in *IKKε*-deficient bristles, suggesting that IKKε regulates the localization but not the expression of Fascin (Fig. 1E,F, Fig. S1C,D). By contrast, Forked was able to localize along actin bundles in *IKKε*-deficient bristles (Fig. 1H,I, Fig. S1E,F). These results suggest that IKKε regulates actin bundling by regulating the localization of Fascin.

To determine the functional relationship between Forked, Fascin and IKKε, we examined the potential genetic interactions between these proteins. The expression of dominant-negative IKKε resulted in a bent and hooked bristle morphology (Fig. 1J) (Oshima et al., 2006; Shapiro and Anderson, 2006; Koto et al., 2009; Bitan et al., 2010; Otani et al., 2011), whereas control bristles did not show any morphological defects (Fig. 1M). Further reduction of *forked* in *IKKε^DN^* bristles significantly exaggerated the bristle morphology phenotype, resulting in frequent bristle branching (Fig. 1L,M), suggesting that IKKε and Forked regulate bristle morphogenesis in a coordinated manner. By contrast, the reduction of *fascin* (*singed*) did not significantly modify the bristle morphology phenotype in *IKKε^DN^* bristles (Fig. 1K,M). Heterozygotes of either *singed^3^* or *forked^36a^* did not show any visible bristle morphology phenotype (Fig. S4A,B). Taken together, these results suggest that IKKε and Forked coordinately regulate Fascin-dependent actin bundling during bristle morphogenesis.

### IKKε regulates the hexagonal packing of actin filaments

**Fig. 2.**
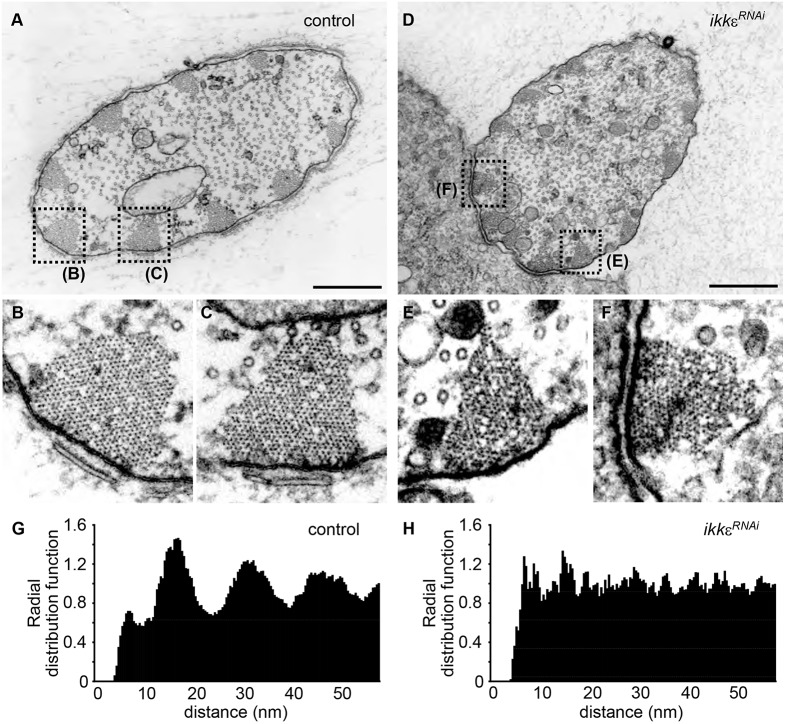
**IKKε regulates the hexagonal packing of actin filaments.** (A-C) TEM analysis of control bristles. Magnified views (B,C) of actin bundles show that the actin filaments are hexagonally packed in a paracrystalline manner. (D-F) TEM analysis of *IKKε^RNAi^* bristles. Magnified views (E,F) of actin bundles show that the actin filament packing is irregular, displaying liquid order. (G) Radial distribution function of control bristles shows multiple distinct peaks, indicating that actin filaments are spaced regularly. (H) Radial distribution function of *IKKε^RNAi^* bristles does not show clear peaks, indicating that actin filament spacing is irregular. Scale bars: 1 µm. See also Fig. S1.

The hallmark of Fascin-dependent actin bundling is the hexagonal packing of actin filaments (DeRosier and Tilney, 1982; Tilney et al., 1998). Transmission electron microscopy (TEM) analysis of the microchaetes on the dorsal thorax revealed that actin filaments are hexagonally packed in a paracrystalline manner in control bristles, indicating that Fascin cross-linking is present in control actin bundles (Fig. 2A-C, Fig. S1G,I) (Tilney et al., 1995). In *IKKε^RNAi^* microchaetes, the size and the number of actin bundles were not severely altered, although the hexagonal packing of the actin filaments was perturbed and the filaments were irregularly packed (Fig. 2D-F, Fig. S1H,J). These results indicate that Fascin-dependent actin bundling is compromised in *IKKε^RNAi^* microchaetes.

The packing pattern of actin filaments was quantitatively evaluated. First, the centroids of actin filaments were determined, and the radial distribution function, which represents the probability of finding other actin filament centroids at a distance *r* from a given reference actin filament centroid, was calculated (Fig. S1K). In control bristles, the radial distribution function showed multiple peaks with 15-16 nm intervals, indicating that actin filaments were regularly spaced (Fig. 2G). The interval size was in agreement with the sum of actin filament diameter and the size of Fascin-dependent cross-links (actin filament diameter of 5-9 nm, Fascin size of ∼8 nm; Alberts et al., 2015; Jansen et al., 2011). By contrast, the radial distribution function of *IKKε^RNAi^* bristles did not yield clear peaks, suggesting that the actin filaments were irregularly spaced (Fig. 2H). Voronoi tessellation analysis also suggested that the hexagonal packing of actin filaments was disorganized in *IKKε^RNAi^* bristles (Fig. S1L-P). These results demonstrate that IKKε regulates the Fascin-dependent formation of paracrystalline actin bundles during bristle morphogenesis.

### Fascin dynamically exchanges in the actin bundles

**Fig. 3.**
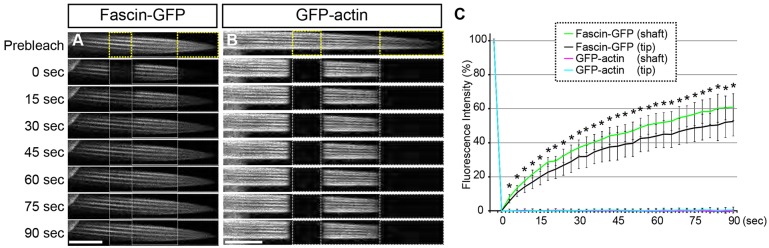
**Fascin dynamically exchanges in actin bundles.** (A) FRAP analysis indicates that Fascin-GFP fluorescence recovered rapidly after photobleaching. (B) By contrast, GFP-actin fluorescence recovery was not observed within this timecourse. Yellow boxes indicate the regions that were photobleached in A and B. (C) Quantification of Fascin-GFP and GFP-actin fluorescence recovery. Fascin-GFP fluorescence recovered rapidly after photobleaching and ∼60% of Fascin-GFP molecules were mobile. The recovery rate was similar for the tip and shaft region. GFP-actin fluorescence did not recover within 90 s. The fluorescence recovery rates are shown as mean±s.d. *n*=4 for Fascin-GFP, *n*=3 for GFP-actin. *P<0.001 by two-tailed *t*-test, Fascin-GFP versus GFP-actin. Scale bars: 10 µm. See also Fig. S3, Movies 1-4.

Fascin has been shown to be a dynamic actin cross-linker that rapidly exchanges within the actin bundles (Vignjevic et al., 2006; Aratyn et al., 2007; Hwang et al., 2015). To examine whether Fascin can dynamically exchange within paracrystalline actin bundles, we examined the dynamics of Fascin in the growing bristles. Fluorescence recovery after photobleaching (FRAP) analysis revealed that Fascin-GFP dynamically exchanges in the actin bundles of growing bristles. (Fig. 3A,C, Movie 1). By contrast, GFP-actin fluorescence recovered little over this timecourse, demonstrating that the actin bundle itself is stably maintained (Fig. 3B,C, Movie 2). These results suggest that Fascin dynamically exchanges within paracrystalline actin bundles during bristle morphogenesis.

### Rab35 does not play a major role in regulating Fascin localization

To elucidate the molecular mechanisms underlying the IKKε-dependent regulation of Fascin, we examined the relationship of IKKε with known regulators of Fascin. Rab35 has been reported to interact with Fascin in a GTP-dependent manner to promote its membrane recruitment to regulate bristle morphogenesis (Zhang et al., 2009). However, contrary to previous observations, overexpression of Rab35[S22N] (GDP-bound form) did not affect bristle morphology (Fig. S2A) or Fascin localization (Fig. S2F) in our experimental conditions. Moreover, although Rab35[Q67L] (GTP-bound form) overexpression partially suppressed the phenotype of *IKKε^DN^* bristles (Fig. S2B-E), it did not restore Fascin localization along the actin bundles (Fig. S2G). These results suggest that IKKε regulates Fascin localization independently of Rab35.

### IKKε regulates Fascin through control of serine 52 phosphorylation

PKC is known to phosphorylate Fascin at a conserved serine residue (serine 39 in mammalian cells, serine 52 in *Drosophila*) to inhibit its actin bundling activity (Yamakita et al., 1996; Ono et al., 1997). A phosphoresistant mutant of Fascin has been shown to possess a PKC-resistant actin bundling activity (Ono et al., 1997), whereas the phosphomimetic mutant of Fascin lacks actin bundling activity (Vignjevic et al., 2006). The phosphomimetic mutant of Fascin, Fascin[S52E], fails to rescue the bristle morphogenesis defects of *Drosophila fascin* mutants, suggesting that this phosphorylation site might be important in bristle morphogenesis (Zanet et al., 2009).

**Fig. 4.**
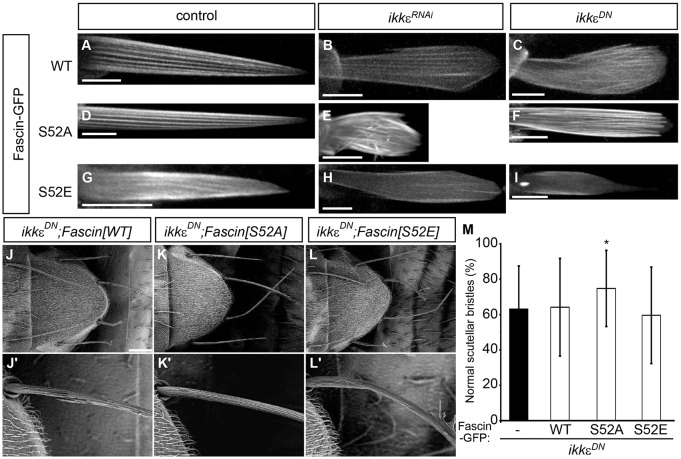
**IKKε regulates Fascin by controlling the phosphorylation status of serine 52.** (A-I) GFP fluorescence images of live pupae expressing Fascin-GFP in bristles. Fascin[WT]-GFP localization along the actin bundles was decreased in *IKKε*-deficient bristles (A-C), whereas Fascin[S52A]-GFP was resistant to the loss of IKKε (D-F). The actin bundle localization of Fascin[S52E]-GFP was reduced (G-I). (J-L′) SEM analysis shows that expression of phosphoresistant Fascin[S52A] suppresses the phenotype of *IKKε^DN^* bristles. Representative bristles are shown at higher magnification in J′-L′. (M) Quantification of the bristle morphology phenotype. The percentage of normal scutellar bristles is shown as mean±s.d. *n*=69 for *IKKε^DN^*, *n*=60 for *IKKε^DN^*/Fascin[WT], *n*=40 for *IKKε^DN^*/Fascin[S52A] and *n*=59 for *IKKε^DN^*/Fascin[S52E]. *P<0.05 by two-tailed *t-*test, compared with *IKKε^DN^*. Scale bars: 10 µm in A-I; 100 µm in J-L. See also Figs S2 and S4.

To determine the relationship between IKKε and PKC-dependent phosphorylation, we expressed wild-type Fascin (Fascin[WT]), phosphoresistant Fascin[S52A] or phosphomimetic Fascin[S52E] in developing bristles. In *IKKε*-deficient bristles, Fascin[WT]-GFP localization along actin bundles was diminished (Fig. 4A-C). By contrast, Fascin[S52A]-GFP was able to localize along actin bundles in *IKKε*-deficient bristles (Fig. 4D-F), whereas the actin bundle localization of Fascin[S52E]-GFP was reduced (Fig. 4G-I). Fascin[S52A]-GFP and Fascin[S52E]-GFP showed similar recovery kinetics compared with Fascin[WT]-GFP in FRAP experiments, although the initial recovery rate of Fascin[S52E]-GFP was more rapid compared with that of Fascin[WT]-GFP and Fascin[S52A]-GFP (Fig. S3). These results suggest that IKKε controls Fascin localization by regulating the serine 52 phosphorylation status. Consistent with this idea, expression of Fascin[S52A]-GFP, but not Fascin[WT]-GFP or Fascin[S52E]-GFP, in *IKKε^DN^* bristles significantly suppressed the bristle morphology defects (Fig. 4J-M), whereas overexpression of any of the three forms in the wild-type background did not affect bristle morphology (Fig. S4C-E). The incomplete suppression of the bristle morphology defects by Fascin[S52A]-GFP in *IKKε^DN^* bristles probably reflects the contribution of other pathways (such as Nuf) operating downstream of IKKε in bristle morphogenesis (Otani et al., 2011). These results indicate that IKKε regulates actin bundling in part by controlling serine 52 phosphorylation of Fascin.

### IKKε protects Fascin from PKC-dependent inhibitory phosphorylation

**Fig. 5.**
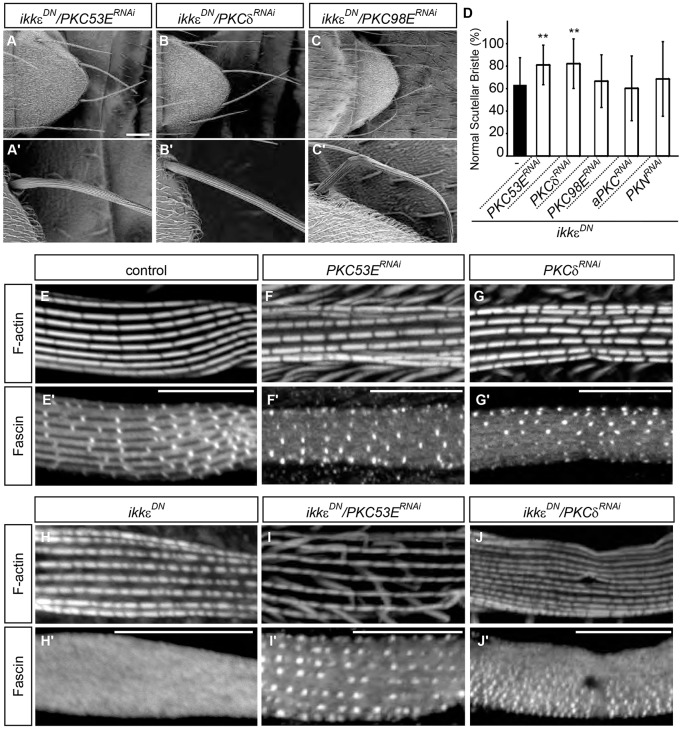
**IKKε regulates Fascin localization by antagonizing PKC.** (A-C′) SEM analysis shows that knockdown of PKC53E or PKCδ suppresses the phenotype of *IKKε^DN^* bristles. Representative bristles are shown at higher magnification in A′-C′. (D) Quantification of the bristle morphology phenotype. The percentage of normal scutellar bristles is shown as mean±s.d. *n*=69 for *IKKε^DN^*, *n*=30 for others. *IKKε^DN^* is identical to Fig. 4M. **P<0.0005 by two-tailed *t-*test, compared with *IKKε^DN^*. (E-J′) Phalloidin (F-actin) and anti-Fascin antibody staining show that knockdown of PKC53E or PKCδ restores the actin bundle localization of Fascin in *IKKε^DN^* bristles. Scale bars: 100 µm in A-C; 10 µm in E-J′. See also Fig. S4.

To determine whether PKC family kinases are involved in IKKε-Fascin signaling, we examined the genetic interactions between IKKε and PKC family genes. Knockdown of either PKC53E (homologous to PKCα/β) or PKCδ led to a significant suppression of the bristle morphology phenotype of *IKKε^DN^* bristles (Fig. 5A,B,D), whereas the knockdown of PKC98E (homologous to PKCε), aPKC or PKN did not modify this phenotype (Fig. 5C,D). Furthermore, knockdown of either PKC53E or PKCδ restored the actin bundle localization of Fascin in *IKKε^DN^* bristles (Fig. 5H-J). Knockdown of PKC family genes in a wild-type background did not affect the actin bundle localization of Fascin (Fig. 5E-G) and did not yield any visible phenotype in bristle morphogenesis (Fig. S4F-H), suggesting that PKC is repressed by IKKε in wild-type bristles. These results suggest that IKKε promotes Fascin-dependent actin bundling in bristle morphogenesis by protecting Fascin from PKC-dependent inhibitory phosphorylation.

### IKKε regulates PKC membrane translocation

**Fig. 6.**
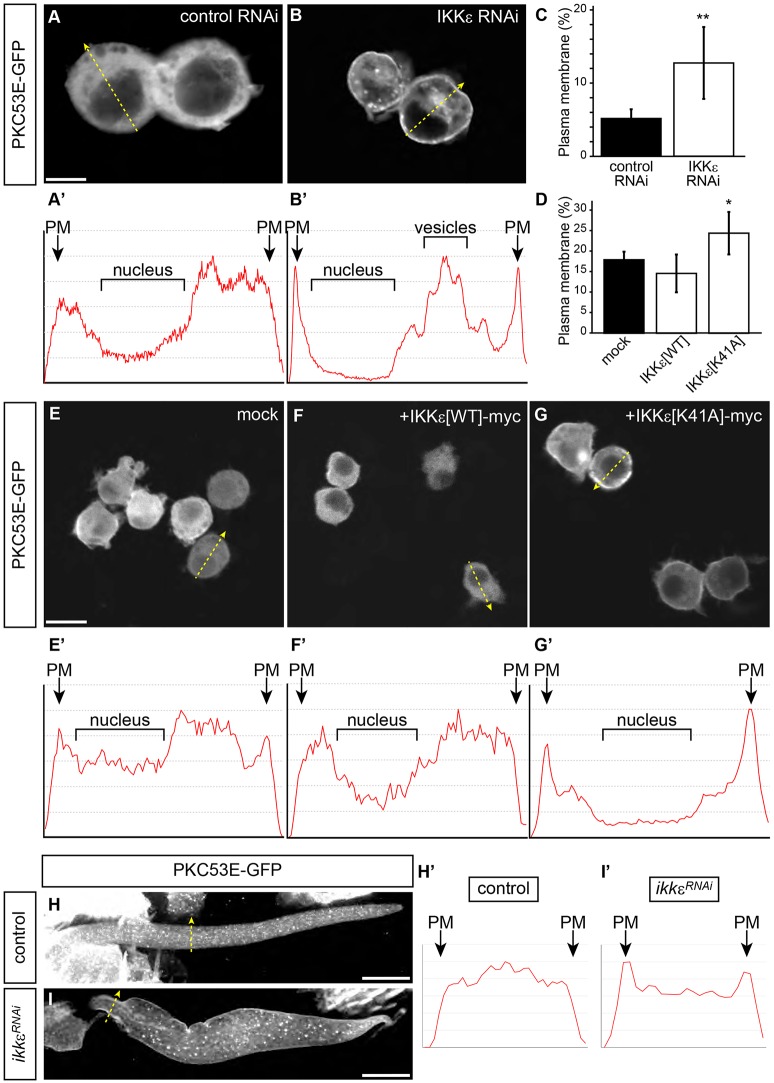
**IKKε inhibits PKC membrane translocation.** (A,B) PKC53E-GFP translocates to the membrane in IKKε-depleted S2 cells. PKC53E is diffusely localized in the cytoplasm in control RNAi (A), but translocates to the membrane in IKKε RNAi (B). (A′,B′) Plot profile along yellow arrows in A,B. (C) Quantitation of plasma membrane localization of PKC53E-GFP in IKKε-depleted S2 cells. **P<0.001 by two-tailed *t-*test, compared with control RNAi. (D) Quantitation of plasma membrane localization of PKC53E-GFP in IKKε-overexpressing S2 cells. *P<0.01 by two-tailed *t-*test, compared with mock. (E-G) IKKε kinase activity is required to inhibit PKC53E-GFP membrane translocation. PKC53E-GFP is enriched in the cytoplasm in mock-transfected (E) or IKKε[WT]-myc-transfected (F) cells, but becomes concentrated at the membrane in IKKε[K41A]-myc-transfected cells (G). Cells with moderate expression level of PKC53E-GFP were examined in A-G. (E′-G′) Plot profile along yellow arrows in E-G. (H,I) IKKε inhibits PKC53E-GFP membrane translocation in developing bristles. (H′,I′) Plot profile along yellow arrows in H,I. PM, plasma membrane. Scale bars: 10 µm. See also Fig. S4.

To gain insight to the molecular mechanisms underlying the crosstalk between IKKε and PKC, we expressed the two molecules in cultured Schneider 2 (S2) cells. We focused on PKC53E for further analysis, as its mammalian homolog PKCα is known to phosphorylate Fascin (Yamakita et al., 1996; Ono et al., 1997). IKKε-myc and PKC53E-GFP interacted with each other and were co-immunoprecipitated from S2 cell lysates (Fig. S5). PKC53E-GFP showed a cytoplasmic localization when expressed in S2 cells (Fig. 6A,E), and coexpression of IKKε[WT] did not alter this localization (Fig. 6F). Interestingly, inhibition of IKKε by RNAi or by expression of dominant-negative IKKε[K41A] resulted in membrane translocation of PKC53E-GFP (Fig. 6B,G). Quantitation confirmed that IKKε inhibits the membrane localization of PKC53E-GFP (Fig. 6C,D). In developing bristles, PKC53E-GFP was localized to the cytoplasm, with some punctate signals in wild-type bristles (Fig. 6H), whereas PKC53E-GFP localized to the plasma membrane and vesicle-like structures in *IKKε^RNAi^* bristles (Fig. 6I). These results demonstrate that IKKε suppresses the membrane localization of PKC53E-GFP.

### IKKε inhibits PKC

Previous studies have shown that newly synthesized PKC is membrane associated but inactive, whereas fully phosphorylated mature PKC localizes to the cytoplasm and can translocate to membranes in response to upstream signals (Newton, 2003). These results suggest that the membrane localization of PKC in IKKε-deficient cells can be due to either defects in PKC maturation or the excess activation of PKC. These two scenarios can be distinguished by examining PKC activity. In the former model, defects in PKC maturation should lead to decreased PKC activity in IKKε-deficient cells, whereas in the latter scenario PKC activity should be increased in IKKε-deficient cells.

**Fig. 7.**
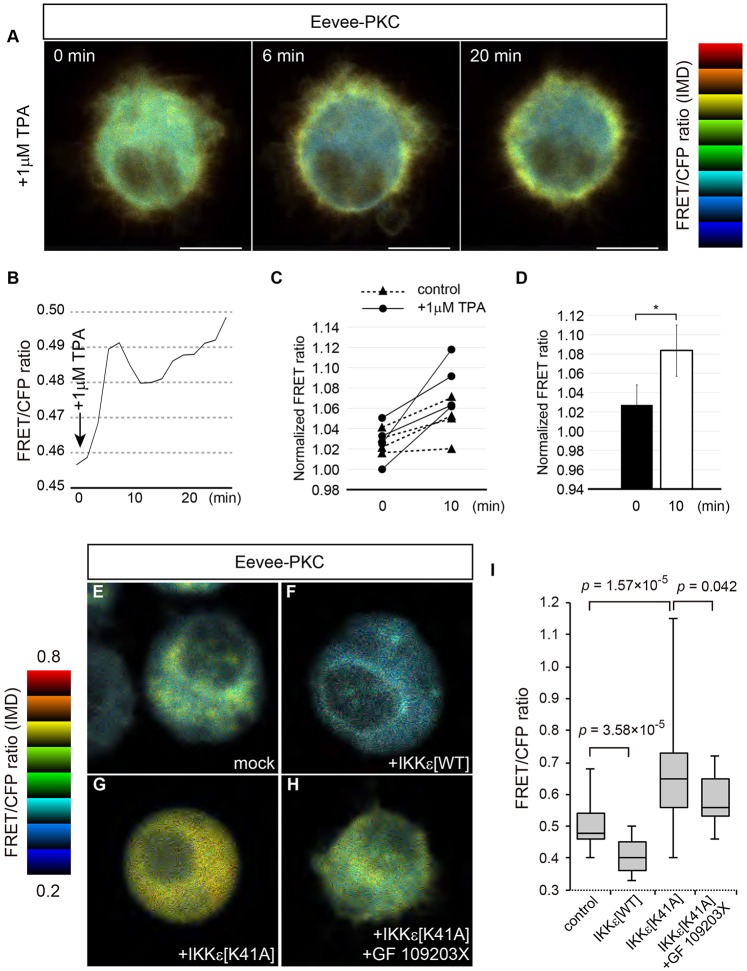
**IKKε inhibits PKC.** (A,B) Eevee-PKC reports PKC activity in *Drosophila* S2 cells. Eevee-PKC-expressing S2 cells were treated with 1 µM TPA, resulting in an acute increase in FRET signals at the membrane. (C,D) Quantification of FRET signals in TPA-treated cells. FRET signal is increased upon addition of TPA. FRET ratio was normalized against the mean FRET ratio prior to the addition of TPA (−10 to 0 min). *P<0.05 by two-tailed paired *t*-test. *n*=4. (E-H) IKKε regulates PKC activity. FRET signal is reduced in IKKε[WT]-expressing cells (F) compared with the control (E), but is increased in IKKε[K41A]-expressing cells (G). Pretreatment with PKC inhibitor (GF 109203X) reduces the FRET signal in IKKε[K41A]-expressing cells (H). (I) Quantification of FRET analysis. *n*=24 for IKKε[K41A], *n*=20 for IKKε[K41A]+GF 109203X, *n*=25 for others. *P*-values determined by the Wilcoxon rank-sum test. IMD, intensity modulated display. Scale bars: 5 µm.

We therefore tested the impact of IKKε on PKC activity by expressing a Förster (or fluorescence) resonance energy transfer (FRET) biosensor for PKC (Eevee-PKC) (Komatsu et al., 2011) in S2 cells. Eevee-PKC is an intramolecular FRET biosensor for PKC, and consists of a donor and acceptor fluorescent protein separated by a phosphate-binding domain, a long and flexible linker, and a PKC substrate peptide. Upon phosphorylation of the substrate peptide by PKC, the biosensor changes its conformation, resulting in an increase in FRET/CFP value (Komatsu et al., 2011). The Eevee-PKC FRET biosensor was able to report PKC activity in S2 cells, as its FRET/CFP signal was increased and enriched in the plasma membrane-associated region upon treatment with the phorbol ester 12-O-tetradecanoylphorbol-13-acetate (TPA) (Fig. 7A-D). FRET analysis revealed that expression of IKKε[WT] suppressed PKC activity (Fig. 7E,F,I), whereas expression of dominant-negative IKKε[K41A] activated PKC (Fig. 7G,I). The cytoplasmic FRET/CFP signals could be due to the diffusion of the FRET biosensor upon prolonged expression of IKKε[K41A]. The increase in FRET/CFP signal in IKKε[K41A]-expressing cells was significantly reduced by pretreatment with the PKC inhibitor bisindolylmaleimide GF 109203X (Toullec et al., 1991), which blocks phosphorylation of the FRET biosensor by PKC (Fig. 7H,I). These results demonstrate that PKC is activated in IKKε[K41A]-expressing cells, supporting the excess activation model. Taken together, these results suggest that IKKε inhibits PKC.

## DISCUSSION

### IKKε prevents excess PKC activation

**Fig. 8.**
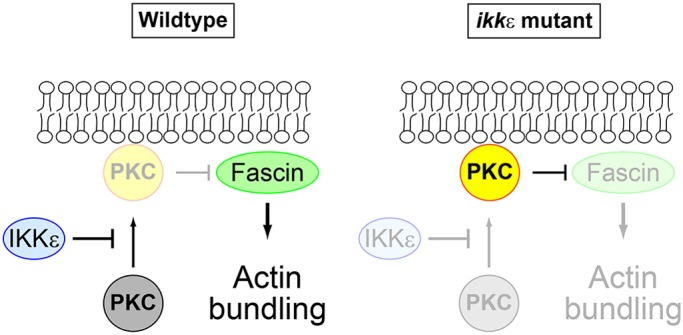
**IKKε prevents excess activation of PKC.** Model for IKKε-dependent regulation of actin bundling. In wild-type bristles, IKKε prevents excess PKC activation, thereby protecting Fascin from inhibitory phosphorylation to promote actin bundling (left). In *IKKε* mutant bristles, excess activation of PKC occurs, resulting in inhibition of Fascin-dependent actin bundling (right).

We have shown that IKKε inhibits PKC so as to protect Fascin from inhibitory phosphorylation, thereby promoting actin bundling during bristle morphogenesis (Fig. 8). PKC is responsible for the inhibition of Fascin-dependent actin bundling in *IKKε*-deficient bristles, whereas knockdown of PKC in a wild-type background yields no visible phenotype. These results suggest that IKKε prevents excess activation of PKC in wild-type bristles. How PKC is activated in *IKKε* mutant bristles remains to be characterized, but it should be noted that several signaling molecules that can potentially activate PKC are known to be involved in pupal thorax development (including Fz, Dpp, PVR) (Adler et al., 1994; Martin-Blanco et al., 2000; Ishimaru et al., 2004). IKKε could act as a switch to activate Fascin-dependent actin bundling by turning off PKC signaling during bristle morphogenesis. Given the widespread involvement of PKC in various signaling pathways, it is likely that IKKε-dependent PKC repression plays important roles in other systems. Of note, gene amplification of *IKKε* has been linked to oncogenesis (Boehm et al., 2007), and PKC functions as a tumor suppressor (Antal et al., 2015). Whether IKKε-dependent suppression of PKC plays any role in oncogenesis remains to be clarified in the future.

PKC membrane translocation and activation is controlled by its interaction with lipids through the C1 and C2 domains (Newton, 2003). Phosphorylation of the C2 domain of the novel PKC in *Aplysia* can reduce its membrane translocation (Farah et al., 2012). Although the molecular mechanisms underlying IKKε-dependent inhibition of PKC remain unknown, it would be interesting to determine whether IKKε can phosphorylate PKC to regulate its membrane translocation and activation.

### Possible roles of Rab35 in bristle morphogenesis

In contrast to previous findings (Zhang et al., 2009), our results suggest that Rab35 does not play a significant role in regulating Fascin localization in bristle morphogenesis. These differences might be due to the different Gal4 drivers used: *prospero-Gal4* in the previous study versus *scabrous-Gal4* in this study. As *prospero-Gal4* drives expression throughout the peripheral nervous system, it is possible that the previously observed bristle morphology defect in Rab35[S22N]-overexpressing flies was due to non-cell-autonomous effects. Nevertheless, overexpression of Rab35[Q67L] can suppress the bristle morphology phenotype of *IKKε*-deficient bristles, which could be due to the involvement of both IKKε and Rab35 in vesicle trafficking (Kouranti et al., 2006; Sato et al., 2008; Otani et al., 2011). Further studies are required to clarify the role of Rab35 in bristle morphogenesis.

### IKKε and actin bundle organization

Our results suggest that IKKε regulates the actin bundling activity of Fascin during bristle morphogenesis. Interestingly, although IKKε is localized at the distal tip of growing bristles (Bitan et al., 2010; Otani et al., 2011, 2015) it can regulate the localization of PKC53E-GFP and Fascin throughout the bristles. As IKKε regulates Fascin via PKC, diffusion of PKC might serve to propagate the inhibitory influence of the tip-localized IKKε signaling on PKC. Our FRAP analysis indicates that Fascin can dynamically exchange within the actin bundles, consistent with the idea that paracrystalline actin bundles are dynamic structures (Hwang et al., 2015). The reversible cross-linking activity of Fascin might allow dynamic regulation of paracrystalline actin bundles during their repair or disassembly (Hwang et al., 2015; Overton, 1967; Tilney et al., 1996).

In addition to its role in regulating actin bundling, IKKε is also required for the cortical localization of actin bundles (Bitan et al., 2010; Otani et al., 2011). Previous studies have demonstrated that Arp2/3 complex-dependent branched actin network structures known as actin ‘snarls' play an important role in localizing the actin bundles to the cell cortex (Hopmann et al., 1996; Hopmann and Miller, 2003; Tilney et al., 2003; Frank et al., 2006). Whether IKKε plays any role in regulating the dynamics of actin snarls, and how distinct actin-based structures interact with each other to organize a parallel array of actin bundles, are important issues to investigate in the future.

## MATERIALS AND METHODS

### *Drosophila* genetics

All *Drosophila* strains were raised at 25°C on standard corn meal agar food. The following strains were used: *y^1^ w^67C21^* as a control; *IKKε^1^* (also known as *IKKε^36^*), *IKKε^alice^* (*IKKε* mutants recombined with FRT40A were from Kathryn Anderson, Sloan Kettering Institute, New York, USA) (Oshima et al., 2006; Shapiro and Anderson, 2006), *y w Ubx-flp;* *ubi-GFP FRT40A/CyO* (*Ubx-flp* from Jürgen Knoblich, IMB, Vienna, Austria) (Xu and Rubin, 1993; Emery et al., 2005), *IKKε^RNAi^* (Oshima et al., 2006), *IKKε^DN^* (Oshima et al., 2006), *sca-Gal4* (Brand and Perrimon, 1993; de Celis et al., 1999), *neuP72-Gal4* (provided by François Schweisguth, Institut Pasteur, Paris, France) (Bellaiche et al., 2001), *forked^36a^* (Petersen et al., 1994), *singed^3^* (Paterson and O'Hare, 1991) (*forked* and *singed* were from Drosophila Genetic Resource Center), *UAS-YFP-Rab35[S22N]*, *UAS-YFP-Rab35[Q67L]* (*UAS-YFP-Rab35* flies were from Bloomington Stock Center) (Zhang et al., 2007), *UAS-Fascin[WT]-GFP*, *UAS-Fascin[S52A]-GFP*, *UAS-Fascin[S52E]-GFP* (*Fascin-GFP* flies were from François Payre, Serge Plaza, and Jennifer Zanet, Université de Toulouse, Toulouse, France) (Zanet et al., 2009), *UAS-PKC53E^RNAi^[TRiP.JF02641]*, *UAS-PKCδ^RNAi^[TRiP.JF02991]*, *UAS-PKC98E^RNAi^[TRiP.JF02470]*, *UAS-aPKC^RNAi^[TRiP.JF01966]* and *UAS-PKN^RNAi^[TRiP.JF02970]* (*UAS-PKC^RNAi^* flies were from Bloomington Stock Center). *UAS-PKC53E-GFP* transgenic flies were generated by standard P-element-mediated transgenesis. See Table S1 for the genotypes used in each experiment.

### Antibodies

The following antibodies were used: mouse anti-Fascin monoclonal (Developmental Studies Hybridoma Bank, clone sn 7c; 1:5) (Cant et al., 1994); rabbit anti-Forked polyclonal antiserum (from Greg Guild, University of Pennsylvania, Philadelphia, USA; 1:500) (Guild et al., 2003); mouse anti-GFP (Roche, clones 7.1 and 13.1, #11814460001; 1:500); rabbit anti-GFP (MBL, #598; 1:500); anti-GFP HRP-DirecT (MBL, #598-7; 1:20,000); and anti-myc HRP-DirecT (MBL, #M047-7; 1:5000). The following secondary antibodies and detection reagents were used: Alexa 488-conjugated anti-mouse IgG (Molecular Probes, #A-11001; 1:200); Alexa 568-conjugated anti-mouse IgG (Molecular Probes, #A-11031; 1:200); Alexa 488-conjugated anti-rabbit IgG (Molecular Probes, #A-11008; 1:200); Alexa 568-conjugated anti-rabbit IgG (Molecular Probes, #A-11011; 1:200).

### Molecular biology

*Pkc53E* cDNA was from the Drosophila Genomics Resource Center (clone GH03188). IKKε[WT] and IKKε[K41A] constructs were characterized previously (Oshima et al., 2006). Fusion constructs were generated by subcloning *Pkc53E* or *IKKε* into pUAST-EGFP-N, pUAST-HA-Ctag, pUAST-myc-Ntag or pUAST-IRES-mKO vectors. The PKC FRET biosensor Eevee-PKC (3599NES) expression vector was provided by Kazuhiro Aoki and Michiyuki Matsuda (Kyoto University, Kyoto, Japan) (Komatsu et al., 2011) and was subcloned into the pUbi-attB vector (Kondo and Hayashi, 2013).

### Immunohistochemistry

Pupae were fixed as described previously (Otani et al., 2011). Blocking was performed in 0.1% BSA, 0.2% Triton X-100 and 0.2% Tween 20 in PBS overnight at 4°C. Anti-Fascin, anti-Forked, mouse anti-GFP, secondary antibodies and Alexa 488/568-conjugated Phalloidin were diluted (as indicated above) in blocking solution, and antibody incubation was performed overnight at 4°C with gentle agitation. Washing with 0.1% Triton X-100 in PBS was performed three times after each antibody incubation step. After the final wash, the thorax specimens were mounted dorsal side up on glass slides in Vectashield mounting medium (Vector Labs) using a cover glass (0.12-0.17 mm thickness, Matsunami) as a spacer.

### Cell culture and immunofluorescence

*Drosophila* S2 cells (provided by Shin-ichi Yanagawa, Kyoto University, Kyoto, Japan) (Schneider, 1972; Yanagawa et al., 1998) were cultured in Schneider's Insect Medium (Gibco) supplemented with 10% FCS and antibiotics at 25°C. Transfection was performed using Effectene (Qiagen) according to the manufacturer's instructions, and cells were harvested 36-48 h after transfection.

For RNAi, dsRNA against *lacZ* (control) or *IKKε* (Oshima et al., 2006) was added to the medium to a final concentration of 37 nM, and 36-48 h later the cells were transferred and transfected with PKC53E-GFP as described above. Cells were harvested 36-48 h after plasmid transfection (3-4 days after dsRNA addition).

For immunofluorescence, cells were fixed in 4% PFA in PBS for 20 min at room temperature, permeabilized with 0.1% Triton X-100 in PBS for 15 min, and blocked with 5% skimmed milk in TBS (Tris-buffered saline). Rabbit anti-GFP primary antibody and secondary antibodies were diluted (as indicated above) in blocking solution, and antibody incubation was performed for 1 h at room temperature. After each antibody incubation, the coverslips were washed three times with 0.1% Triton X-100 in PBS. The cells were mounted in Vectashield mounting medium.

### Confocal microscopy

Confocal microscopy was performed using an FV1000 laser scanning confocal microscope mounted on a BX61 microscope using a UPlanSApo 60×/NA 1.35 objective or UPLSAPO 40×2/NA 0.95 objective (all Olympus) and argon (488 nm) and helium-neon (543 nm) lasers. Macrochaetes were examined for all of the samples, and image acquisition was performed using Fluoview software (Olympus). Fascin-GFP still images were obtained from living samples without fixation, and were captured using an FV1000 laser scanning confocal microscope mounted on an IX81 inverted microscope with a PlanApo N 60×/NA 1.42 objective (all Olympus) and an argon (488 nm) laser, and image acquisition was performed using Fluoview software. A Gaussian blur filter was applied to the acquired stacked images. PKC53E-GFP images from macrochaetes and Fascin/F-actin images in *PKC^RNAi^* bristles were obtained using a TCS-SPE laser scanning confocal microscope mounted on a DMI4000B inverted microscope with an HCX PL APO 63×/NA 1.40 objective (all Leica) and diode laser lines (488/532 nm), and image acquisition was performed using LAS AF software (Leica). Image processing (*z*-stacking, Gaussian blur filter application, brightness and contrast adjustments and generation of plot profiles) were performed using ImageJ (NIH) or Photoshop (Adobe) without any nonlinear adjustments. *z*-stacks of Fascin immunostaining were generated by maximum intensity projections of cortical sections. PKC53E-GFP plasma membrane localization was quantified by measuring the ratio between plasma membrane intensity (obtained by line scan) and total intensity of individual cells using ImageJ. Cells that express PKC53E-GFP at relatively low levels were analyzed to avoid artifacts caused by overexpression. Statistical significance was evaluated by *t*-test (two-tailed) using Excel (Microsoft).

For FRAP analysis, pupal cases were removed with fine forceps and the pupae were placed dorsal side down in glass-bottom dishes (Iwaki). Two pieces of 3MM filter paper were placed lateral to the pupae, and water was added to cover the pupae to avoid drying. Images of pupae were captured using the FV1000 mounted on an IX81 with the PlanApo N 60×/NA 1.42 objective and argon (488 nm) laser. GFP fluorescence was captured at 3 s intervals with *z*-sectioning. Photobleaching was performed using a diode (405 nm) laser. Fluorescence intensity of the selected region of interest (ROI) was measured using ImageJ and normalized by setting the pre-bleach level to 100% and the post-bleach level (0 s after photobleaching) to 0%. Statistical significance was evaluated by *t*-test (two-tailed) using Excel.

For FRET analysis, S2 cells were cotransfected with pUbi-Eevee-PKC, actin5Ce-Gal4 driver, and pUAST-IRES-mKO or pUAST-IKKε[WT]-IRES-mKO or pUAST-IKKε[K41A]-IRES-mKO, and plated on glass-bottom dishes. mKO-expressing cells were imaged using the FV1000 mounted on an IX81 with the PlanApo N 60×/NA1.42 objective. FRET probe was excited by a diode laser (440 nm), and the ratio between the CFP and FRET signals was calculated using ImageJ. The intensity modulated display (IMD) ratio images were generated using an in-house-written ImageJ plugin (developed by Housei Wada). For TPA treatment, TPA (Sigma) was added to the culture medium at 1 µM final concentration and immediately imaged. FRET ratio was normalized against the mean FRET ratio prior to the addition of TPA (−10 to 0 min), and statistical significance was evaluated by paired *t*-test (two-tailed) using Excel. For GF 109203X hydrochloride (Sigma) treatment, cells were pretreated with 30 nM or 300 nM GF 109203X prior to transfection. The GF 109203X concentration did not affect the results, and data for 30 nM treatment are presented. Multiple comparisons of FRET ratio were performed by Kruskal–Wallis test, and *P*-values for pairwise comparisons were calculated using the Wilcoxon rank-sum test using R statistical software.

### Scanning electron microscopy (SEM)

Adult flies were anesthetized with CO_2_ and their legs and wings were removed using fine forceps. The dissected flies were mounted dorsal side up, sputter-coated with platinum (JFC-1600, JEOL) or osmium (Neoc-STB, Meiwafosis), and viewed by SEM (JSM-5600-LV, JEOL) under low vacuum (30 Pa) using an acceleration voltage of 10 kV.

### Transmission electron microscopy

Pupal cases were removed with fine forceps and the pupae dissected in fixative (2.0% glutaraldehyde in 0.05 M phosphate buffer, pH 6.8; as described by Tilney and Tilney, 1994). The dorsal thorax explant was then transferred to fresh fixative. Pupae were fixed at room temperature for 2-4 h and then transferred to 4°C. After three 10 min washes with 0.05 M phosphate buffer (pH 6.8) on ice, pupae were postfixed with 1% OsO_4_ in 0.05 M phosphate buffer (pH 6.8) for 45 min on ice. The fixed pupae were then washed three times for 10 min in water and stained *en bloc* with 0.5% aqueous uranyl acetate overnight on ice. After washing for 5 min with water, pupae were dehydrated in a graded acetone series (25, 50, 70, 80, 90, 95 and 99.5%) for 15 min at each concentration and transferred to 100% acetone for two 20 min incubations. Polybed 812 (Polysciences) or Quetol 651 (NEM) was used as the resin. When Polybed 812 was used, pupae were incubated in propylene oxide for 20 min twice, infiltrated with a 1:1 mixture of propylene oxide:Polybed 812, and finally placed into 100% Polybed 812. The resin was polymerized for more than 48 h at 60°C. For Quetol 651, pupae were incubated in a 1:1 mixture of n-butyl glycidyl ether (QY1):acetone for 20 min, incubated in 100% QY1 for 20 min, infiltrated with a 1:1 mixture of QY1:Quetol 651 for 2 h, and finally placed into 100% Quetol 651. The resin was polymerized for more than 48 h at 60°C. Semi-thin (0.4-0.7 µm) sections were cut from the anterior side of the pupae and examined using Toluidine Blue staining to confirm orientation. Based on the appearance of the dorsal thoracic microchaetes, the orientation of the pupae was carefully readjusted to obtain a perfect transverse section of the bristle. After confirming orientation, ultrathin sections (∼60 nm) were cut and mounted on 50-mesh Formvar-coated copper grids. The sections were stained with 4% aqueous uranyl acetate in the dark for 10 min, stained with Reynold's lead citrate for 3 min, and then coated with a thin layer of carbon. Observations were performed using a JEM-1010 transmission electron microscope (JEOL) at 100 kV accelerating voltage, and images were captured by a Gatan Bioscan 792 digital camera.

### Quantification of hexagonal packing of actin filaments

To quantify the hexagonal packing of actin filaments, a Gaussian blur filter was applied to the original TEM images to reduce noise, and binary images of the centroids of the actin filaments were generated with the Find Maxima command of ImageJ. The image was subsequently subtracted with a binary image generated from the Gaussian-filtered image by thresholding to reduce erroneous detection of actin filament centroids. Radial distribution functions were measured by calculating the density of actin filaments centroids within a distance of *r* and *r*+*dr* away from a given centroid. Calculation was performed for *r*=0.6-59.4 nm with *dr*=1.2 nm and Δ*r*=0.4 nm. The centroids used for the measurement were manually selected so that the actin filament centroids at the perimeter of the bundles were not included within the 60 nm diameter. Data from all measurable centroids were averaged to obtain the final radial distribution. Voronoi tessellation was performed using the Voronoi commands of ImageJ. The shapes of the polygons were analyzed using in-house-written programs (available upon request). Statistical analyses by two-tailed *t*-test were performed using Excel.

### Quantification of the bristle phenotype

Bristle morphology was examined using a Stemi 2000 dissection microscope (Zeiss) and the phenotype of the scutellar bristles was scored. The number and morphology of the scutellar bristles of each animal were recorded, and bristles with a branched, hooked, bifurcated or bulged morphology were classified as ‘abnormal'. The mean percentage of normal bristles was calculated, and two-tailed *t*-test performed using Excel. All data are presented as mean±s.d.

### Biochemistry

Transfected S2 cells were lysed in lysis buffer (50 mM Tris-HCl pH 7.5, 150 mM NaCl, 0.5% Triton X-100, 10% glycerol, 1 mM EDTA, 1 mM DTT). The lysates were incubated for 30 min at 4°C, then cleared by centrifugation at 20,000* ****g*** for 10 min at 4°C. 20 µl anti-GFP beads (MBL) were added to the supernatant and the samples incubated with rotation for 2 h at 4°C. The beads were rapidly washed three times with lysis buffer, and the complexes were eluted by boiling in 2×Laemmli sample buffer supplemented with 10% β-mercaptoethanol. SDS-PAGE was performed by standard methods using 5-20% SuperSep Ace polyacrylamide gels (Wako). Western blotting was performed as described previously (Otani et al., 2011). Anti-GFP HRP-DirecT and anti-myc HRP-DirecT were diluted (as indicated above) in Can Get Signal (Toyobo).
